# Melittin inhibits osteosarcoma growth *in vitro* and *in vivo* by inactivating the Wnt/β-catenin signaling pathway

**DOI:** 10.7150/jca.109750

**Published:** 2025-07-28

**Authors:** Xiaobo Wang, Junli Chang, Xingyuan Sun, Suxia Guo, Yimian Li, Qi Shi, Yanping Yang

**Affiliations:** 1 Longhua Hospital, Shanghai University of Traditional Chinese Medicine, Shanghai, 200032, China.; 2 Key laboratory of theory and therapy of muscles and bones, Ministry of Education, Shanghai, 200032, China.

**Keywords:** melittin, osteosarcoma, Wnt/β-catenin, tumor proliferation, apoptosis

## Abstract

**Objective:** Osteosarcoma is a highly aggressive primary bone tumor that predominantly affects pediatric and adolescent populations. This study aims to explore the therapeutic potential and underlying mechanisms of melittin in treating osteosarcoma both *in vitro* and *in vivo*.

**Materials and methods:** Two osteosarcoma cell lines, namely 143B and U-2 OS, were utilized to assess the impact of melittin on cellular proliferation, apoptosis, and cell cycle progression. The effect of melittin on cell viability was evaluated using the CCK-8 assay. Flow cytometry was employed to assess apoptosis and cell cycle progression, while Western blotting analyzed the expression of key proteins, including Cdc-2, c-Myc, and ꞵ-catenin. Specifically, we employed small interfering RNA (siRNA) to selectively knock down the expression of β-catenin in two osteosarcoma cell lines. In *vivo* studies, tumor growth in nude mice was evaluated through measurements of tumor weight, volume. Additionally, immunohistochemical analysis was performed to assess Ki-67 and active ꞵ-catenin expression in tumor tissues.

**Results:** Melittin induced apoptosis in 143B and U-2 OS osteosarcoma cells in a concentration-dependent manner and caused S-phase cell cycle arrest as the drug concentration increased. Mechanistic investigations revealed that melittin's efficacy was associated with the inactivation of the Wnt/ꞵ-catenin signaling pathway. Specifically, melittin treatment reduced the expression of phosphorylated glycogen synthase kinase-3 beta (p-GSK-3ꞵ) and active ꞵ-catenin. Notably, silencing β-catenin in 143B and U-2 OS osteosarcoma cells significantly enhanced the anti-proliferative and pro-apoptotic effects of melittin. This was evidenced by reduced cell viability and increased apoptosis, thereby further confirming that the anti-tumor efficacy of melittin is dependent on the inhibition of the Wnt/β-catenin signaling pathway. *In vivo* studies confirmed that melittin significantly inhibited the growth of subcutaneously implanted tumors in nude mice. This inhibition was further supported by Ki-67 analyse and H&E staining, while immunohistochemical analysis revealed an obvious reduction in active ꞵ-catenin expression.

**Conclusions:** Our results offer deeper mechanistic insights into the inhibitory impact of melittin on osteosarcoma progression, at least in part by suppressing the Wnt/β-catenin signaling pathway. We expand upon previous research by providing more comprehensive and robust evidences that underscore the potential of melittin as a viable and safe therapeutic agent for osteosarcoma.

## Introduction

Osteosarcoma is one of the most common malignant bone tumors in children and adolescents, characterized by poor prognosis and high mortality rate 1. Osteosarcoma primarily arises in the growth plate of the metaphysis of long bones, such as the femur, tibia, and humerus 2-4. Advances in clinical practice, including timely surgical intervention and the use of cytotoxic chemotherapy regimens, have increased the five-year overall survival rate to approximately 70% 5, 6. However, distant metastasis remains a significant challenge, resulting in a precipitous decline of the five-year overall survival rate to less than 30%, or even lower 7, 8. The current standard treatment strategy, which combines multi-drug chemotherapy with surgery, has undeniably improved limb-sparing rates and extended patient survival 9, 10. Despite these advancements, the treatment outcomes for patients with metastasis, recurrence, or multi-drug resistance remain suboptimal 11-13. Furthermore, the non-selective cytotoxic effects of chemotherapy on healthy cells introduce additional uncertainty regarding treatment efficacy and patient quality of life. These limitations highlight an urgent need for the development of more effective and less toxic therapeutic agents to provide alternative and improved options for osteosarcoma management.

Natural products have long been recognized as a reliable and high-quality source for anti-cancer drug development 14. Bee venom, synthesized and secreted by the venom glands of honeybees, contains melittin as a major active peptide component, comprising approximately 40-55% of its total dry weight 15. Melittin exhibits a broad spectrum of biological effects within the body 16-18. Studies have shown that melittin possesses remarkable therapeutic efficacy against various cancers, including lung cancer, breast cancer, and glioblastoma, through mechanisms such as inducing tumor cell apoptosis and necrosis, inhibiting cellular proliferation, and disrupting the cell cycle 19-21. In recent years, interest in the pharmaceutical potential of melittin for osteosarcoma treatment has grown 22. However, the pharmacological effects and underlying mechanisms by which melittin mediates its anti-osteosarcoma activity remain insufficiently understood, warranting further investigation.

The Wnt/β-catenin signaling pathway is known to play a critical role in organogenesis, stem cell renewal, and numerous other crucial cellular processes 23, 24. Aberrant regulation of this pathway is closely associated with tumorigenesis and its progression, making it a key focus in cancer research and treatment development 25, 26. ꞵ-catenin, a central component of the Wnt/ꞵ-catenin signaling pathway, functions as a transcriptional co-activator. The stabilized, unphosphorylated ꞵ-catenin subsequently translocates from the cytoplasm into the nucleus, where it accumulates and interacts with nuclear transcription factors such as T-cell factor/lymphoid enhancer-binding factor (TCF/LEF). This interaction leads to the transcriptional activation of key target genes, including *c-Myc*, *cyclin D1*, and *Cdkn1a*, which are integral to cell proliferation and survival 27-29.

In this study, we comprehensively investigated the therapeutic potential of melittin in inhibiting osteosarcoma growth *in vitro* and *in vivo* using osteosarcoma cell lines 143B and U-2 OS. Subsequently, we further explored the underlying mechanism of melittin's action, with a focus on the inactivation of the Wnt/β-catenin signaling pathway. We provided strong evidence for the osteosarcoma therapeutic properties of melittin by using siRNA-mediated β-catenin knockdown, highlighting its promising potential as a therapeutic agent for osteosarcoma.

## Materials and Methods

### Compounds and reagents

MEM medium and RPMI-1640 medium (Hyclone, Utah, USA); Melittin peptide (Chinapeptides, Shanghai, China); Doxorubicin (DOX, Sangon Biotech, Shanghai, China); Dimethyl sulfoxide (DMSO, Invitrogen, Rockville, USA); Fetal Bovine Serum (FBS) and Penicillin-Streptomycin (P-S). (Gibco, Grand Island, USA), RIPA cell lysis buffer, SDS-PAGE gel preparation kit, bicin choninic acid (BCA) protein concentration determination kit; Western Blot antibodies against GSK-3β (Cat No : 22104-1-AP) and p-GSK-3β (Cat No : 14850-1-AP) (Proteintech, Wuhan, China); Western Blot antibody against active β-catenin (Cat No : 8814S) and Cdc2 (Cat No :77055S) (Cell Signaling Technology, Danvers, USA); Western Blot Anti-β-actin antibody (Cat No : SAB5600204) (Sigma-Aldrich, St. Louis, USA); Immunohistochemical antibody against active ꞵ-catenin (Cat No: 8814S) (Cell Signaling Technology, Danvers, USA); Ki-67 cell proliferation detection kit (Cat No : KGA9402-50) (KeyGEN BioTECH, Jiangsu, China); Signal silencing (R) β-catenin siRNA (GenePharma, Shanghai, China); Lipofectamine® 2000 transfection reagent (Thermo Fisher scientific Inc, Rockford, USA).

### Cells and culture

The human osteosarcoma cell lines 143B and U-2 OS were provided by the American Type Culture Collection (ATCC, Manassas, USA), while MC3T3-E1 cells were procured from the Cell Bank of the Chinese Academy of Sciences (CAS, Shanghai, China). 143B cells were cultured in MEM medium, whereas U-2 OS cells were maintained in RPMI-1640 medium containing 10% FBS and 100U/mL penicillin and streptomycin(P-S). All osteosarcoma cells were incubated at 37°C in a 5% CO_2_ atmosphere.

### Cell transfection

The 143B and U-2 OS cells were co-transfected with small interfering RNA (si-RNA) targeting β-catenin (100 nM si-β-catenin) or 100 nM negative control (NC) for 48 hours in the presence of Lipofectamine 2000 transfection reagent.

### Measurement of cell viability

Cell viability was assessed using the Cell Counting Kit-8 (CCK-8, Dojindo, Shanghai, China) assay. Briefly, cells were seeded in 96-well plates at a density of 5 × 10^3^ cells/100µL/well under various experimental conditions. After a specified incubation period, the culture medium in each well was replaced with 100 µL of CCK-8 solution (1:9 ratio of CCK-8 to serum-free medium). The plate was incubated at 37℃ for 2 hours in the dark, and cell viability was determined by measuring the absorbance at 450 nm using a Bio-Tek microplate reader.

### Apoptosis detection by flow cytometry

143B and U-2 OS cells were cultured in 6-well plates at a concentration of 1 ×10^5^/ml for 24 hours. Following a starvation period at 37℃ for 12 hours, cells were treated with various drugs. Apoptosis was detected using the Annexin V-FITC and propidium iodide Apoptosis Detection Kit (BD Biosciences, Bedford, USA) according to the manufacturer's instructions. Briefly, cells were incubated with FITC-labeled Annexin V for 30 minutes at room temperature in the dark, followed by staining with propidium iodide on ice for 5 minutes. Flow cytometry was performed to analyze early and late apoptosis, and the apoptosis rate was calculated.

### Cell cycle determination by flow cytometer

143B and U-2 OS cells were seeded in 6-well plates at a density of 1 × 10^5^/ml for 24 hours. Following a starvation period at 37℃ for 12 hours, cells were treated with different drugs for an additional 24 hours. Cell cycle distribution was analyzed using a cell cycle assay kit (Key GENBioTECH, Nanjing, China) according to the manufacturer's instructions. Cells were fixed overnight in 70% cold ethanol and then incubated with RNase A at 37℃ for 30 minutes. After staining with propidium iodide on ice for 5 minutes, flow cytometry was performed to assess cell cycle distribution. The percentage of cells in the G0/G1, S, and G2/M phases was determined using ModFit Software.

### Western blotting analysis

After drug intervention, 143B and U-2 OS cells were washed twice with pre-chilled PBS. RIPA lysis buffer was added, and the lysates were centrifuged at 13,000g for 15 minutes at 4℃. The protein concentrations of the extracted samples were measured using the bicinchoninic acid (BCA) method. Proteins were separated via SDS-PAGE, with the gel concentration selected based on the molecular weight of the target protein, and subsequently transferred onto PVDF membranes. The membranes were incubated overnight at 4℃ with primary antibodies diluted appropriately, followed by three washes and incubation with HRP-conjugated secondary antibodies (Beyotime Institute of Biotechnology, Shanghai, China). Protein bands were visualized using High-sig ECL substrate.

### *In vivo* model establishment and treatment

Animal experiments were conducted under the supervision and approval of the Animal Protection and Utilization Committee of Shanghai University of Traditional Chinese Medicine (PZSHUTCM200731014). Four-week-old BALB/c nude mice (Sippr-BK, Shanghai, China), weighing 16-18g, were housed in SPF-grade isolation cages at the Animal Experiment Center of Shanghai university of traditional Chinese medicine. 143B osteosarcoma cells were digested with trypsin-EDTA, and mixed with Matrigel to prepare a suspension at a concentration of 2 × 10⁷ cells/ml. A 10 µl aliquot of this suspension was subcutaneously injected into the axilla of each mouse to establish an osteosarcoma xenograft model. The mice were randomly assigned to three groups: saline (saline-treated negative control) group, melittin (melittin-treated) group, DOX (doxorubicin-treated positive control) group (n=10 per group).

Twenty-four hours post-injection, mice in the saline group received 100 µl of saline containing DMSO (1/10,000 V/V), the melittin group received 100 µl melittin (1 mg/kg body weight), and the DOX group received 100 µl doxorubicin (1 mg/kg body weight). Treatments were administered via intratumoral injection once daily. Tumor volume and body weight were recorded every three days. Tumor volumes calculated using the formula: volume = (a × b^2^)/^2^, where *a* is the long diameter and *b* is the short diameter. After 19 days of treatment, the mice were euthanized, tumors were excised, weighed, and imaged.

### Immunohistochemistry (IHC)

Tumor tissues were fixed in 10% buffered paraformaldehyde for 24 hours, embedded in paraffin, and sectioned into 5 µm slices. The sections were deparaffinized, rehydrated with PBS (pH 7.4), and treated with 3% hydrogen peroxide to quench endogenous peroxidase activity. After antigen retrieval, sections were blocked with 5% BSA for 30 minutes at room temperature, followed by overnight incubation at 4℃ with primary antibodies. The following day, sections were treated with a secondary antibody at room temperature for 15 minutes and stained using diaminobenzidine (DAB) substrate. Hematoxylin was used as a counterstain, and the sections were mounted with neutral gum after dehydration. We captured images using an Olympus SLIDEVIEW VS200 microscope and quantified the area ratio of Ki-67-positive cells and the staining intensity of active β-catenin in tumor tissue using ImageJ software. To prevent errors resulting from image acquisition, we standardized bright-field microscopy illumination settings as consistently as possible. Specifically, for quantifying the area ratio of Ki-67-positive cells, we applied the threshold tool to segment positive cells in at least three regions of interest per group, ensuring the elimination of non-specific staining. We then calculated the fraction of positive cell areas relative to the total area. Additionally, for detecting active β-catenin expression intensity, we used ImageJ software for background subtraction and color deconvolution to isolate specific staining signals from non-specific background noise. The integrated optical density (IOD) and mean gray value were measured in at least three random regions of interest. A higher IOD value indicates stronger staining intensity. Finally, all obtained data were statistically analyzed using GraphPad Prism software for quantitative evaluation.

### Hematoxylin and Eosin (H&E) Staining

Tumor tissues were fixed in 10% buffered paraformaldehyde for 24 hours, embedded in paraffin, and sectioned into 5 µm thick slices. The sections were stained with H&E and visualized using an Olympus SLIDEVIEW VS200 microscope.

### Statistical analysis

Data from at least three independent experiments were presented as mean ± standard deviation (SD). Statistical analyses were performed using SPSS 23.0, and graphs were generated with Graphpad 7.01 software. Statistical significance was denoted as follows: *P<0.05, **P<0.01, and ***P<0.001.

## Results

### Melittin inhibited osteosarcoma cell viability

The inhibitory effect of melittin on osteosarcoma cell viability was evaluated using the CCK-8 assay in two osteosarcoma cell lines, 143B and U-2 OS. Cells were treated with melittin at concentrations ranging from 200 nM to 1000 nM for 48 hours. DMSO (1/10,000 V/V) was used as the negative control, while doxorubicin (DOX, 0.5 μM) served as the positive control.

**Figure 1:**
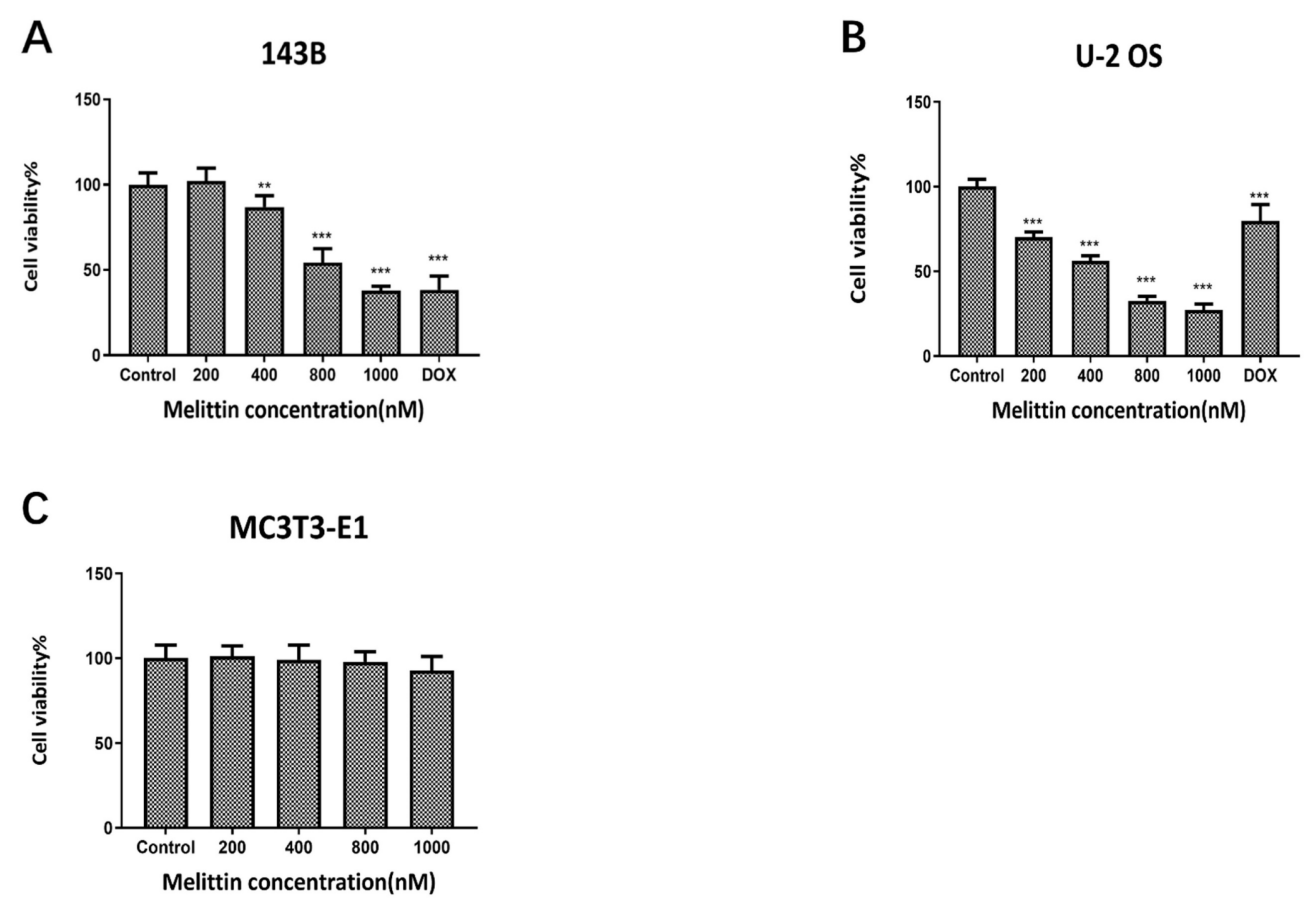
Effect of melittin on the viability of osteosarcoma cells as determined by the CCK-8 assay. Cell viability was assessed in 143B cells (A), U-2 OS cells (B) and MC3T3 (C) cells. **P<0.01, ***P<0.001 *vs.* Control. Control: negative control group.

Cell viability was determined by measuring absorbance at 450 nm using a Bio-Tek ELISA reader, and the IC50 values were calculated to quantify the inhibitory effects. As shown in Figure 1A and Figure 1B, melittin significantly reduced the viability of 143B and U-2 OS cells in a concentration-dependent manner. In contrast, no notable inhibitory effect was observed in mouse preosteoblastic MC3T3-E1 cells (Figure 1C), suggesting that melittin selectively targets osteosarcoma cells while sparing normal bone cells.

### Melittin induced apoptosis in osteosarcoma cells

Following the demonstration of melittin's inhibitory effect on osteosarcoma cell viability, we examined whether this effect was associated with apoptosis induction. 143B and U-2 OS cells were treated with melittin at concentrations of 200 nM, 400 nM, and 800 nM for 24 hours, while the control group received dimethyl sulfoxide (DMSO) at a concentration of 1/10,000 V/V.

**Figure 2:**
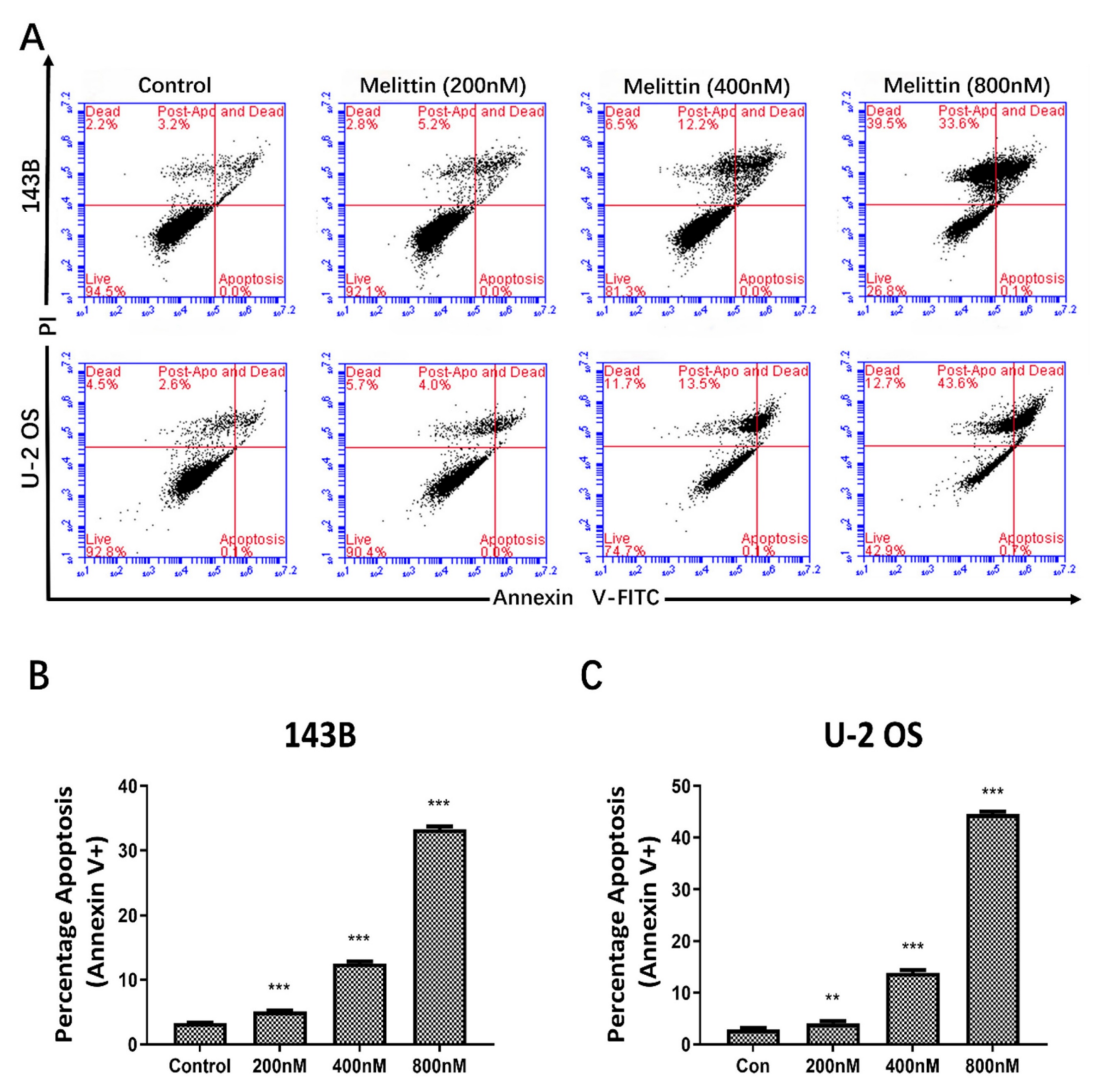
Effect of melittin on apoptosis of osteosarcoma cells as confirmed by Flow Cytometry. (A) Flow cytometry analysis of apoptosis in osteosarcoma cell lines 143B and U-2 OS following treatment with varying concentrations of melittin. Histograms (B) and (C) depict the percentage of apoptotic cells in 143B and U-2 OS osteosarcoma cells from three independent experiments. **P<0.01, ***P<0.001 *vs.* Control. Control: negative control group.

Flow cytometry analysis revealed that melittin induced apoptosis in a concentration-dependent manner in both 143B and U-2 OS cells. As illustrated in Figures 2A-2C, the apoptosis rates of both 143B and U-2 OS cells exhibited a significant increase as the concentration of melittin was elevated. These results confirm that the growth-inhibitory effects of melittin are, at least in part, mediated by the induction of apoptosis.

### Melittin induced cell cycle arrest at the S phase in osteosarcoma cells

To further investigate the impact of melittin on cell cycle progression in osteosarcoma cells, we exposed 143B and U-2 OS cells to melittin at concentrations of 200 nM, 400 nM, and 800 nM for 24 hours. DMSO was used as a control intervention at a concentration of 1/10000 V/V. Subsequently, flow cytometry analysis was conducted to assess the effect of melittin on cell cycle distribution in osteosarcoma cells.

**Figure 3:**
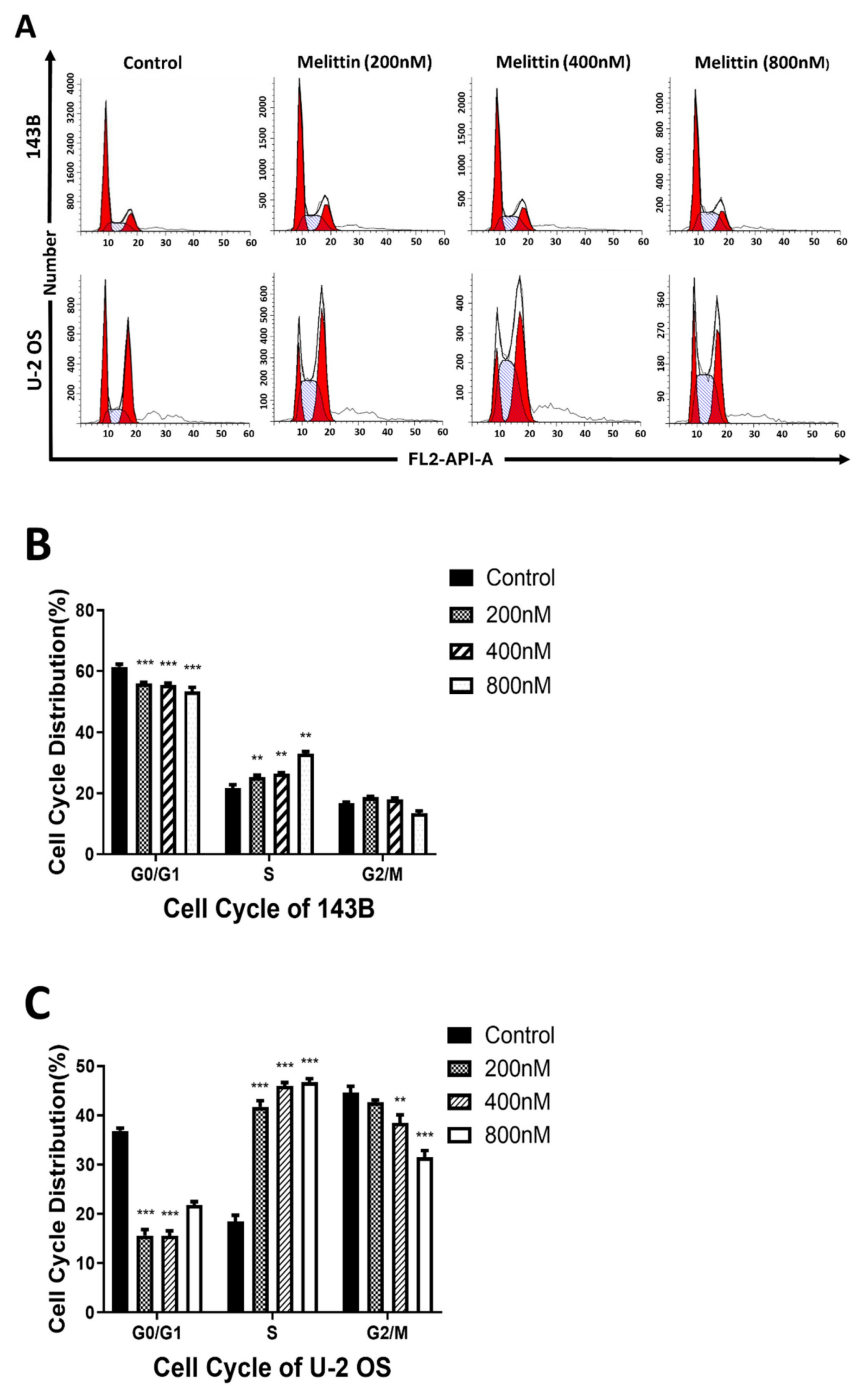

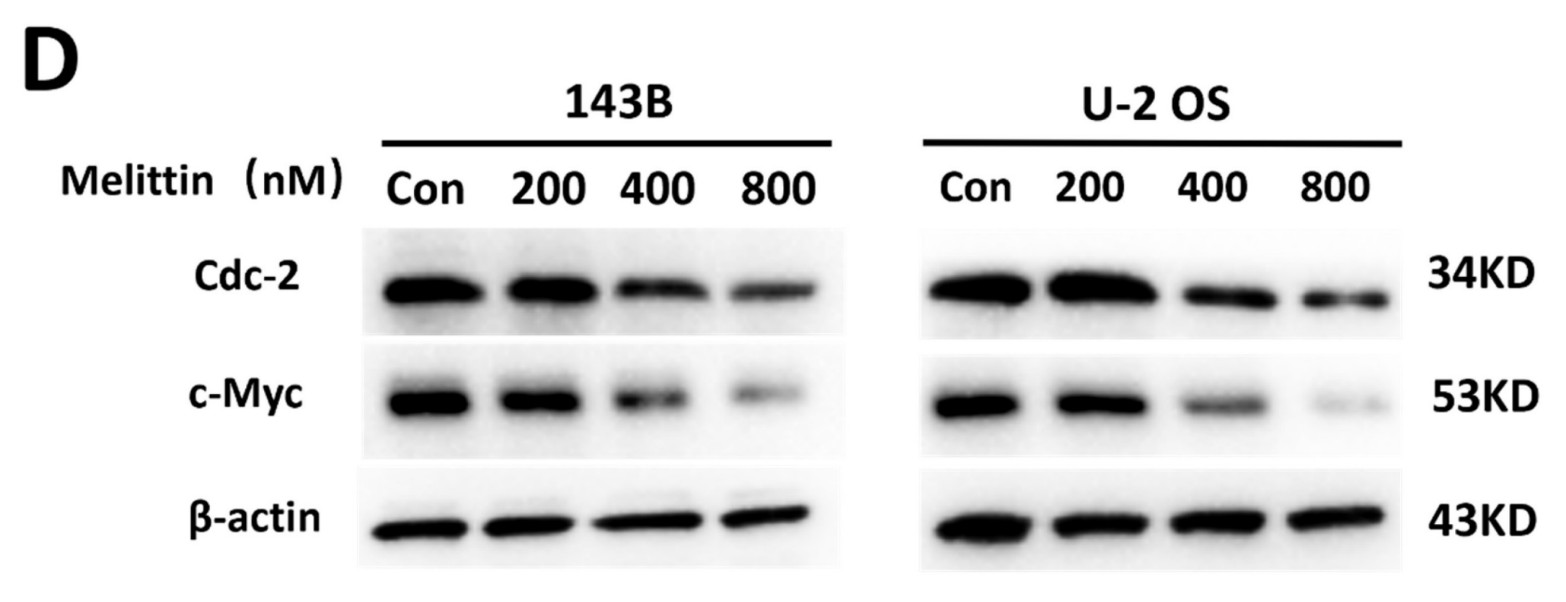
Effect of melittin on cell cycle distribution in osteosarcoma cells. (A) Representative flow cytometry plots depicting the distribution of specific cell cycle phases in osteosarcoma cell lines 143B and U-2 OS following treatment with varying concentrations of melittin. DNA Content Histogram distribution by cell cycle statistical analysis of osteosarcoma cells 143B (B) and U-2 OS (C) treated with different concentrations of melittin. (D) Western blot analysis was employed to evaluate the expression levels of* C-Myc* and *Cdc-2* in 143B and U-2OS cells following treatment with varying concentrations of melittin for 24 hours. β-actin was used as a loading control. **P<0.01, ***P<0.001 *vs.* Control. Control: negative control group.

As shown in Figure 3A-3C, melittin treatment resulted in a concentration-dependent arrest of the cell cycle in the S phase in both 143B and U-2 OS cells. Additionally, Western blot analysis revealed a significant downregulation of Cdc-2 and c-Myc protein expression in both cell lines following melittin treatment (Figure 3D). These findings suggest that melittin effectively disrupts cell cycle progression and inhibits DNA synthesis in osteosarcoma cells, contributing to its anti-tumor effects.

### Melittin induced inactivation of Wnt/β-catenin signaling pathway

The above experiments confirmed that melittin downregulated the expression of c-Myc protein, a downstream target of the Wnt/ꞵ-catenin signaling pathway. Given this connection, we hypothesized that melittin inhibits osteosarcoma cell viability by modulating this pathway. Furthermore, as a critical upstream regulator, GSK-3ꞵ effectively suppresses β-catenin phosphorylation levels.

**Figure 4:**
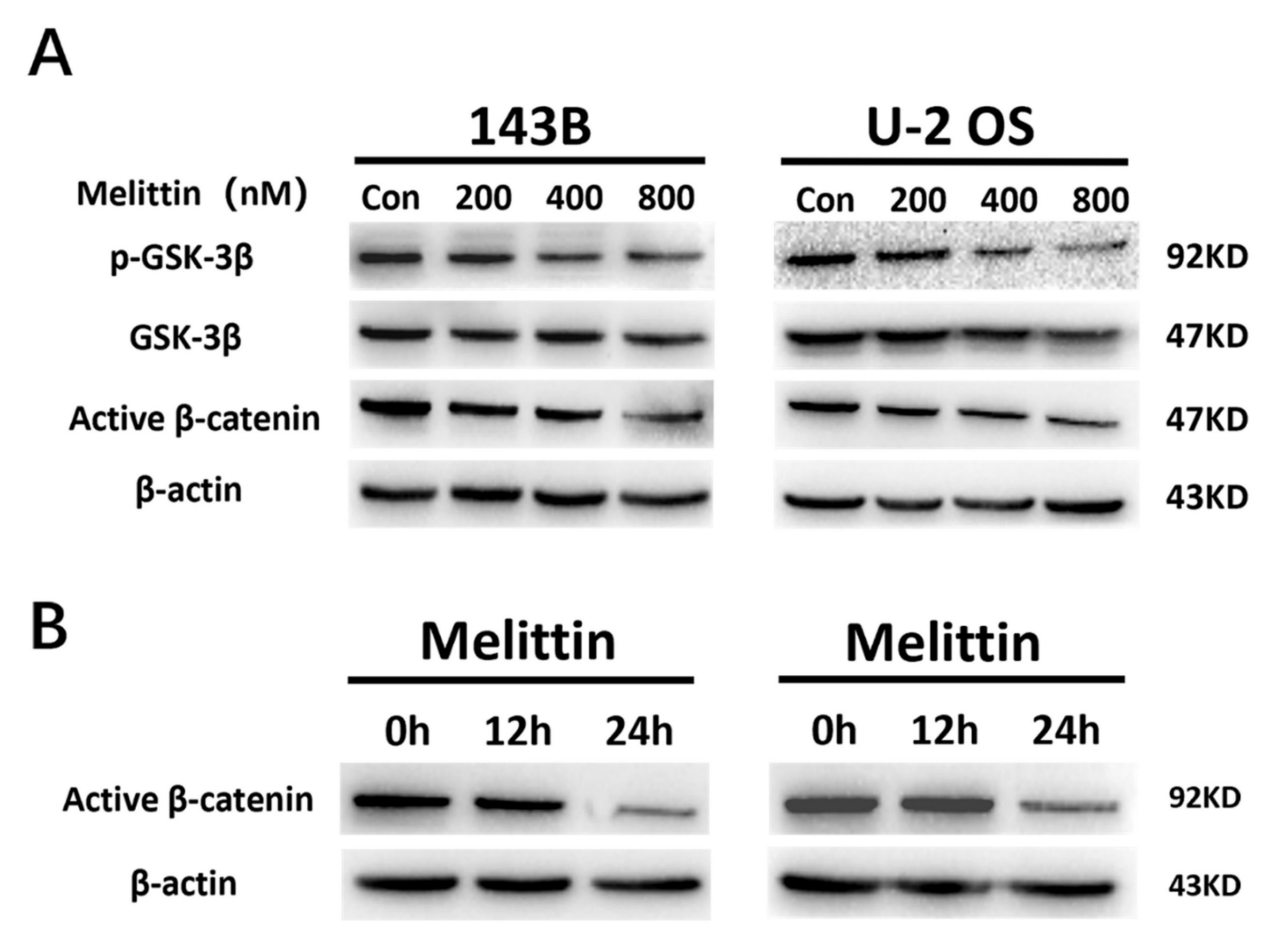
Melittin inhibited the activation of the Wnt/β-catenin signaling pathway. (A) Expression of key proteins in 143B and U-2 OS cells treated with varying concentrations of melittin for 24 hours. (B) Time-dependent effects of melittin on key protein expression. Protein levels were analyzed by Western blotting, with β-actin serving as a loading control.

To further investigate the impact of melittin on the β-catenin signaling pathway in osteosarcoma cells, we treated 143B and U-2 OS cells with concentrations of 200 nM, 400 nM and 800 nM for 24 hours. Subsequently, we employed Western blotting analysis to examine active β-catenin levels along with GSK-3β and phosphorylated GSK-3β (p-GSK-3β) proteins within this pathway, using β-actin as a loading control. The results revealed that melittin induced a concentration-dependent reduction in p-GSK-3β expression and concurrently decreased active β-catenin levels (Figure 4A).

Additionally, a time-course experiment was performed using 800 nM melittin, with cells harvested at 12 and 24 hours. The results demonstrated a time-dependent inhibition of active β-catenin protein expression by melittin (Figure 4B).

These results suggest that melittin effectively inactivates the Wnt/β-catenin signaling pathway, which may contribute to its inhibitory effects on osteosarcoma development.

### Melittin inhibited osteosarcoma development by specifically inactivating the Wnt/β-catenin signaling pathway

To elucidate the crucial role of Wnt/β-catenin signaling pathway in suppressing osteosarcoma cell proliferation and to further investigate the therapeutic mechanism of melittin, CCK-8 and apoptosis assays were conducted to β-catenin-silenced osteosarcoma cells.

143B and U-2 OS cells were transfected with small interfering RNAs targeting ꞵ-catenin (siRNA-ꞵ-catenin) for 48 hours. Transfection efficiency was validated using RT-PCR, ensuring suitability for subsequent experiments. The cells were then treated with 800 nM melittin and divided into four experimental groups: control, si-ꞵ-catenin, melittin, and si-ꞵ-catenin + melittin. Cell viability and apoptosis rates were measured across all groups.

**Figure 5:**
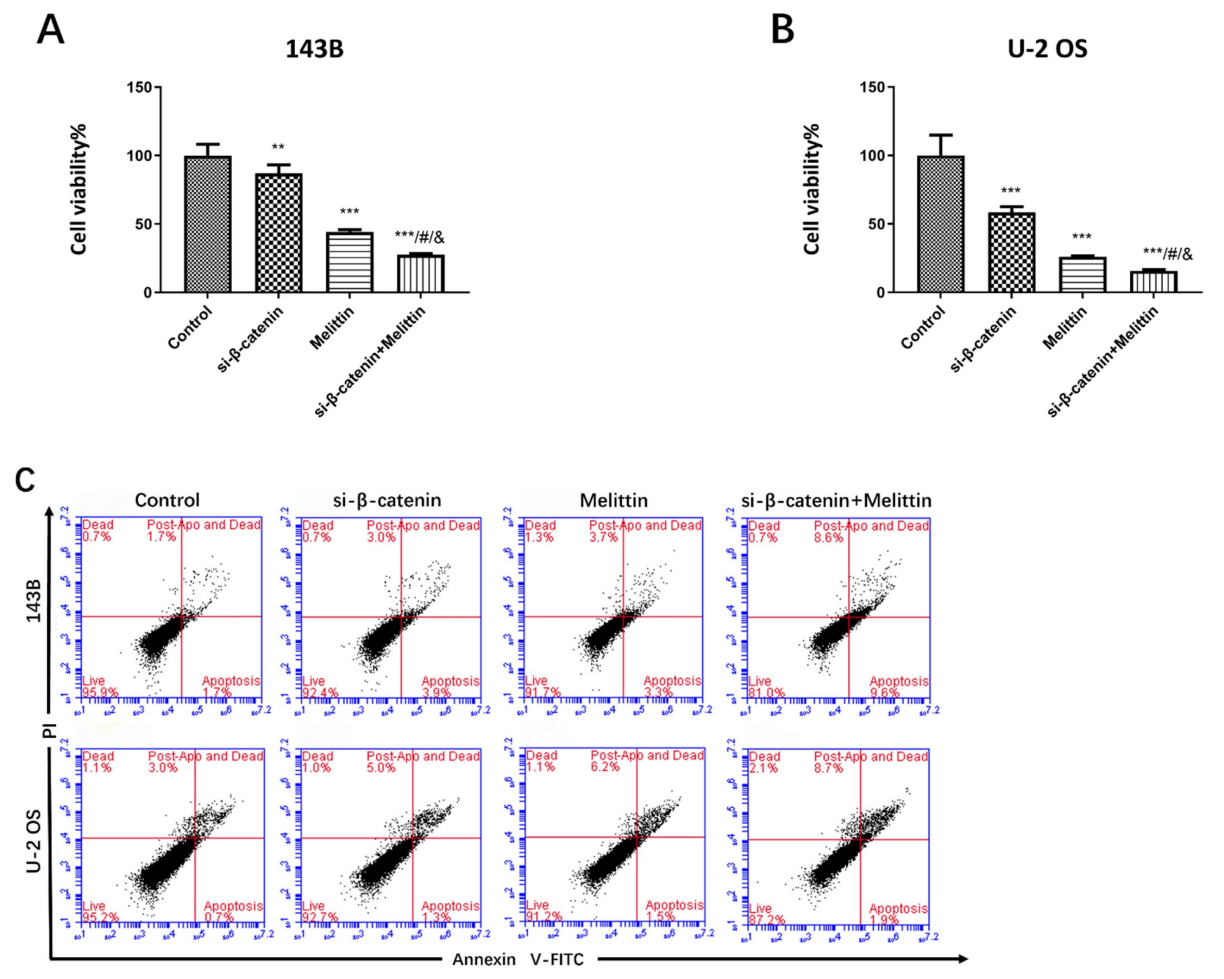

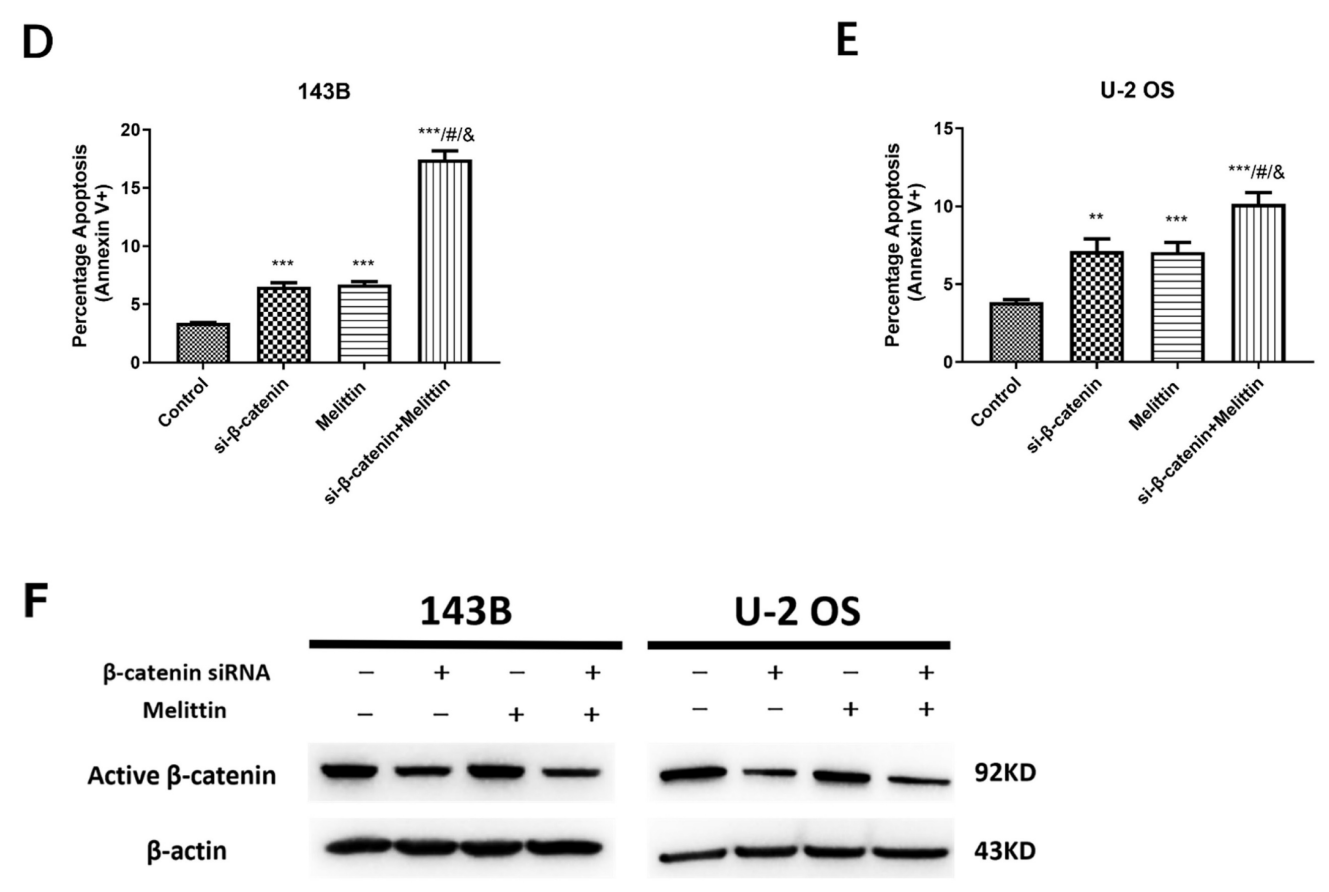
Melittin inhibited proliferation and induced apoptosis in osteosarcoma cells through specific inactivation of β-catenin signaling. Cell viability (A, B), apoptosis (C-E) and active ꞵ-catenin protein expression (F) in 143B and U-2 OS cells treated with 800 nM melittin, either alone or combined with ꞵ-catenin silencing via small interfering RNA (siRNA-ꞵ-catenin). **P<0.01, ***P<0.001 *vs.* the control; #*P*<0.001, si-β-catenin + 0.8 μM melittin *vs.* si-β-catenin; & *P*<0.001, si-β-catenin + 0.8 μM melittin *vs.* 0.8 μM melittin. Control: negative control group.

As shown in Figure 5A-B, ꞵ-catenin silencing combined with melittin treatment resulted in a significant reduction in the survival rates of both 143B and U-2 OS cells compared to cells treated with melittin alone or those with ꞵ-catenin silencing alone. Additionally, a marked increase in apoptosis rates was observed in the combination treatment group (Figure 5C-E).

Moreover, Western blotting analysis demonstrated that active β-catenin protein expression was significantly reduced in the combination treatment group compared to the melittin-only group (**Figure 5F**).

These findings suggest that melittin may inhibit proliferation and promote apoptosis in osteosarcoma cells through specific inactivation of the Wnt/β-catenin signaling pathway.

### Melittin inhibited tumor development in xenograft mice *in vivo*

To further explore the effect of melittin on osteosarcoma progression *in vivo*, we utilized a xenograft model by injecting 143B cells subcutaneously into 4-week-old nude mice. Doxorubicin (DOX, 1 mg/kg body weight) served as the positive control and was administered directly to the tumor site three times per week. Tumor volumes and body weights were monitored every 3 days, while other analyses were performed at the end of the 19-day treatment period when the mice were sacrificed.

**Figure 6:**
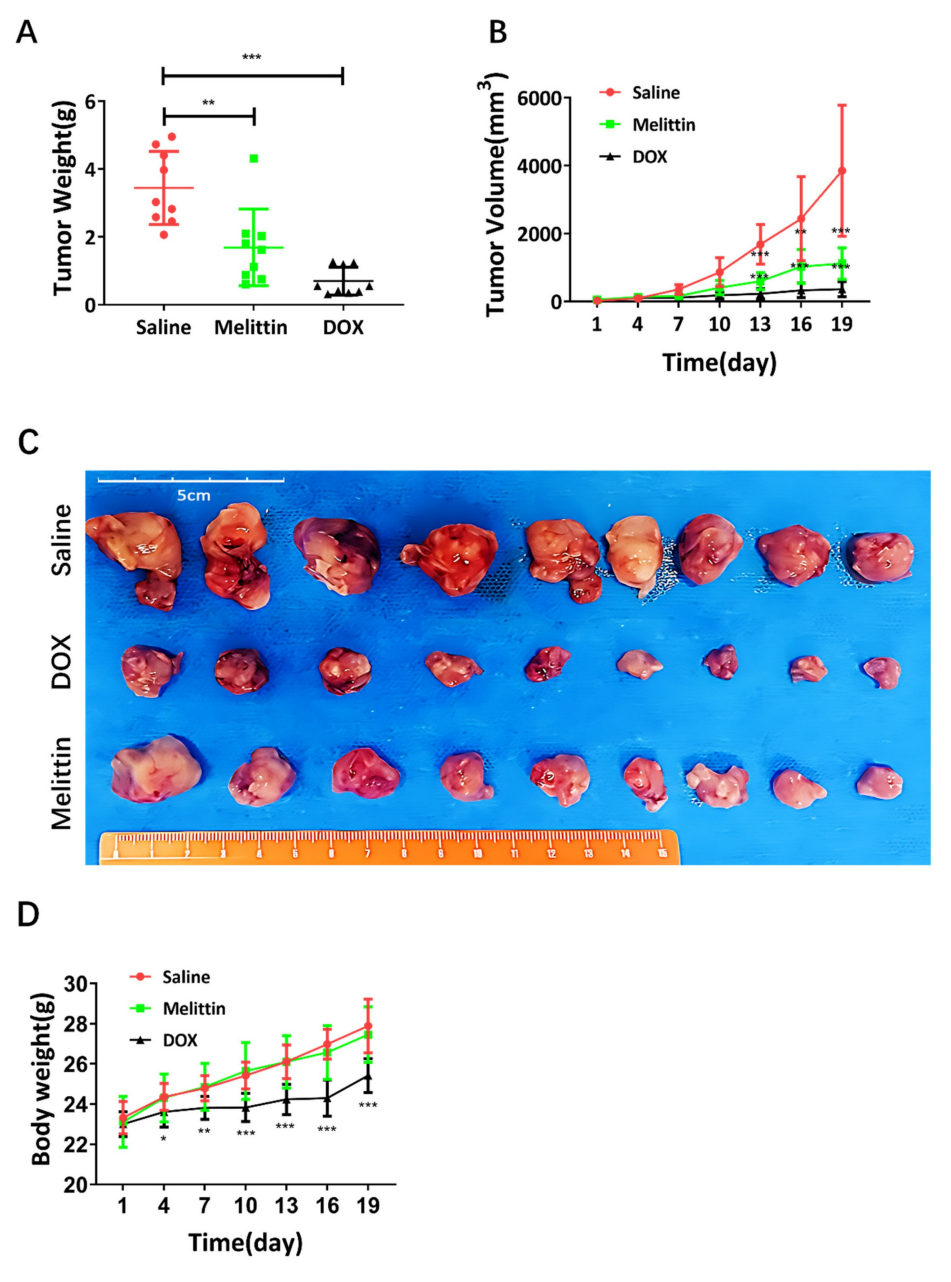

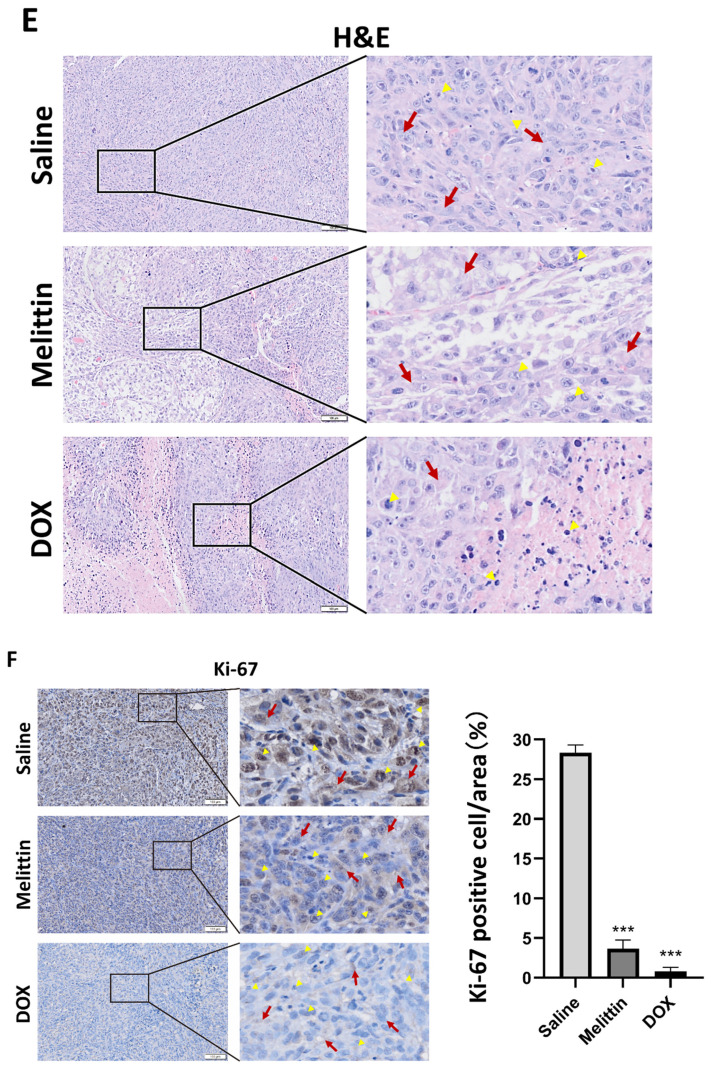

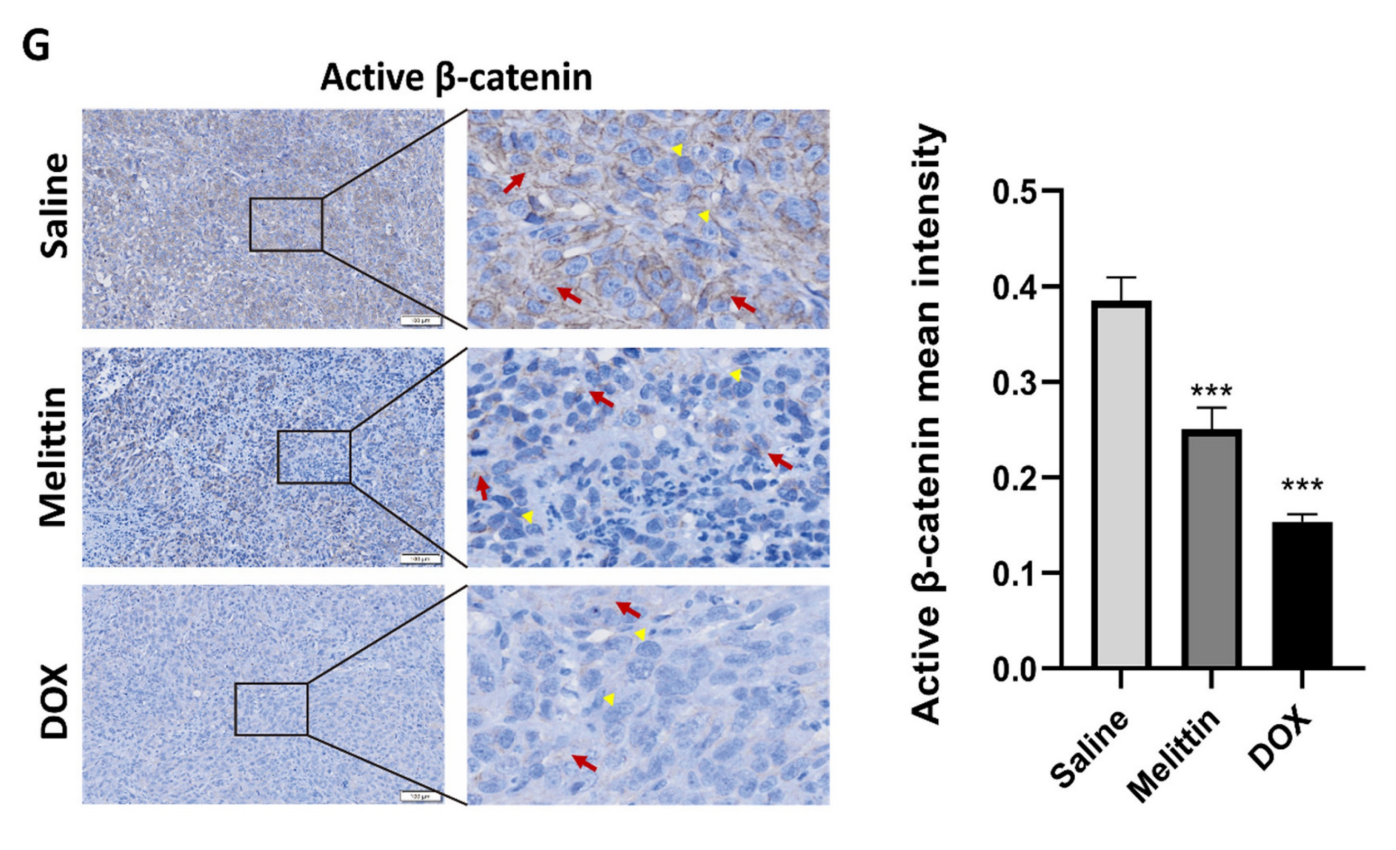
Melittin inhibited osteosarcoma development in tumor-bearing nude mice via subcutaneous injection of 143B cells. Differences in tumor weight and volume were observed and documented in the melittin group compared to the control and positive control groups, as shown in Figures (A) and (B), respectively. (C) Depicts the tumor morphology of xenografts from three groups of human osteosarcoma models, (D) The body weights of mice in the three groups were monitored and recorded. (E) HE staining, the red arrows indicate the cytoplasm of the osteosarcoma cells, while the yellow arrowheads point to the nuclei. (F) Ki-67 immunohistochemical staining, the red arrows indicate proliferating osteosarcoma cells, while the yellow arrowheads highlight osteosarcoma nuclei. The bar graph illustrates the variation in the area ratio of Ki-67-positive cells across different groups. (G) Active β-catenin immunohistochemical staining. The red arrows indicate cytoplasmic expression of active β-catenin in osteosarcoma cells within the tumor body, while the yellow arrowheads point to nuclear localization, and the bar graph illustrates the variation in staining intensity of active β-catenin within tumor tissues. Images were acquired using an OLYMPUS SLIDEVIEW VS200 microscope at 200 × magnification. Saline: saline-treated negative control group; Melittin: melittin-treated group; DOX: doxorubicin-treated positive control group. *P<0.05, **P<0.01, ***P<0.001 *vs.* Saline group.

Melittin treatment significantly reduced both tumor weight (Figure 6A) and tumor volume (Figure 6B) compared to the saline negative control group. Tumors in the melittin group were visibly smaller than those in the saline negative control group, although some differences were observed when compared to the DOX-treated positive control group (Figure 6C). Importantly, no significant differences in body weight were noted between the melittin-treated and saline negative control groups, indicating minimal systemic toxicity (Figure 6D).

Histological examination via H&E staining revealed that melittin treatment resulted in a decreased nuclear-to-cytoplasmic ratio, as well as increased karyopyknosis and karyorrhexis in cells, compared with the saline-negative control group (Figure 6E). Moreover, Ki-67 immunohistochemistry staining revealed a marked decrease in brown nuclear staining in tumors from the melittin-treated group compared to the saline negative control group (Figure 6F), suggesting that melittin significantly suppresses tumor cell proliferation. Furthermore, immunohistochemical analysis showed a significant reduction in active β-catenin expression in melittin-treated tumors (Figure 6G). These results collectively indicate that melittin effectively inhibited osteosarcoma development *in vivo* by suppressing cell proliferation and targeting the Wnt/β-catenin signaling pathway.

## Discussion

Osteosarcoma, a highly aggressive malignant bone tumor, commonly manifests with local swelling, persistent pain, and restricted mobility. Pathologically, it is characterized by osteolytic bone destruction, rapid proliferation, early metastasis, and significant heterogeneity 30-32. While melittin, the primary component of bee venom, has been shown to induce apoptosis, regulate the cell cycle, inhibit tumor cell migration and invasion, and disrupt tumor-associated angiogenesis, its precise mechanisms of action in osteosarcoma remain incompletely understood 33, 34.

Tumorigenesis is closely linked to uncontrolled cell proliferation and evasion of apoptosis 35. In this study, CCK-8 assays revealed that melittin effectively inhibited osteosarcoma cell growth in a concentration-dependent manner, while sparing normal osteoblastic MC3T3 cells. This selective inhibition underscores melittin's therapeutic potential. Apoptosis, often suppressed during tumorigenesis, is critical for cancer control 36. Our findings demonstrated that melittin promotes apoptosis in osteosarcoma cells in a dose-dependent manner, suggesting its potential role in restoring apoptotic pathways. Furthermore, melittin induced S-phase cell cycle arrest, accompanied by downregulation of key cell cycle regulators, such as Cdc-2 and c-Myc, corroborating its growth-inhibitory effects. The downregulation of ꞵ-catenin and its downstream target, c-Myc, suggests that melittin exerts its anti-tumor effects by deactivating the Wnt/ꞵ-catenin signaling pathway. Therefore, in order to investigate the therapeutic value of melittin targeting β-catenin, we employed siRNA-mediated knockdown of β-catenin. The results demonstrated that the anti-tumor efficacy of melittin was significantly enhanced following β-catenin knockdown. Specifically, melittin decreased the levels of active β-catenin in a time- and concentration-dependent manner by inhibiting GSK-3β phosphorylation, thereby promoting β-catenin degradation. Notably, the downregulation of active β-catenin and its downstream target c-Myc was consistently observed in both cell lines, underscoring the pivotal role of this pathway. These results align with reports highlighting the role of GSK-3ꞵ in ꞵ-catenin ubiquitination and proteasomal degradation 37. Cytological evidence motivated us to further investigate the *in vivo* anti-tumor efficacy of melittin and its underlying mechanisms using a nude mouse xenograft model. Compared to controls, melittin significantly reduced tumor volume, weight, and Ki-67 expression, indicating potent anti-proliferative activity. Furthermore, a notable inhibitory effect of melittin on the expression of active β-catenin was observed in the tumor tissues. Importantly, melittin exhibited minimal systemic toxicity, as evidenced by stable body weight in treated mice, reinforcing its therapeutic feasibility.

Emerging research has established the critical role of the Wnt/ꞵ-catenin signaling pathway in tumorigenesis 38-40. Our current work significantly advances previous studies 41, 42. In addition to the 143B cells, we have included U-2 OS cells in our current study to provide a more solid and comprehensive evaluation of melittin's effects in osteosarcoma. This is extremely important due to the inherent heterogeneity of cancer cells, therefore enhances the applicability and generalizability of our results. To further elucidate the role of Wnt/β-catenin pathway in mediating melittin's effects, we employed siRNA-mediated β-catenin knockdown. The results provided strong, mechanism-based evidence that melittin inhibits growth and promotes pro-apoptotic effects in osteosarcoma cells with β-catenin silencing, clearly establishing β-catenin as a key mediator of melittin's activity. Notably, GSK-3ꞵ serves as a critical regulatory enzyme upstream of β-catenin. Our results also offer more in-depth mechanistic insights into the effects of melittin treatment on osteosarcoma. Moreover, the tumor-selective action of melittin in osteosarcoma cells and its *in vivo* safety have not been previously reported. To evaluate the safety of melittin both *in vitro* and *in vivo*, we initially assessed its effects on osteosarcoma cell lines (143B and U-2 OS) as well as normal osteoblasts. The results demonstrated that melittin selectively targets osteosarcoma cells while sparing normal bone cells. Furthermore, the body weight of osteosarcoma xenograft mice in the treatment group remained stable, providing additional evidence for the safety of melittin in an osteosarcoma model.

Although this study provided valuable insights into the anti-tumor effects and molecular mechanism of melittin in osteosarcoma, several limitations warrant consideration. While our findings demonstrated that melittin inhibits osteosarcoma growth by inactivating the Wnt/ꞵ-catenin signaling pathway, the upstream regulators and other potentially interacting pathways affected by melittin were not explored. Given the multifactorial nature of tumor progression, it is likely that additional signaling pathways contribute to the observed effects. Moreover, although body weight remained stable in melittin-treated mice, a comprehensive toxicity assessment-encompassing the impact on vital organs, hematological parameters, and long-term safety-was not conducted.

In conclusion, melittin inhibits osteosarcoma growth by deactivating the Wnt/ꞵ-catenin signaling pathway, selectively targeting tumor cells while sparing normal bone cells. Furthermore, we conducted an in-depth validation of the comprehensive mechanism by which melittin targets the Wnt/β-catenin signaling pathway for osteosarcoma treatment. This work will facilitate subsequent preclinical studies aimed at evaluating its safety, efficacy, and detailed mechanisms of action.
